# Is Zooplankton an Entry Point of Microplastics into the Marine Food Web?

**DOI:** 10.1021/acs.est.3c02575

**Published:** 2023-07-27

**Authors:** Kuddithamby Gunaalan, Torkel Gissel Nielsen, Rocío Rodríguez Torres, Claudia Lorenz, Alvise Vianello, Ceelin Aila Andersen, Jes Vollertsen, Rodrigo Almeda

**Affiliations:** † National Institute of Aquatic Resource, Technical University of Denmark, Kemitorvet, 201, 2800 Kgs. Lyngby, Denmark; ‡ Department of the Built Environment, Aalborg University, Thomas Manns Vej 23, 9220 Aalborg East, Denmark; § Laboratoire d’Océanographie de Villefranche sur mer (LOV), UPMC Université Paris 06, CNRS UMR 7093, Sorbonne Université, 06230 Villefranche sur Mer, France; ∥ EOMAR-ECOAQUA, University of Las Palmas of Gran Canaria, 35017 Las Palmas de Gran Canaria, Spain

**Keywords:** microplastics, zooplankton, copepods, ingestion, fecal pellets

## Abstract

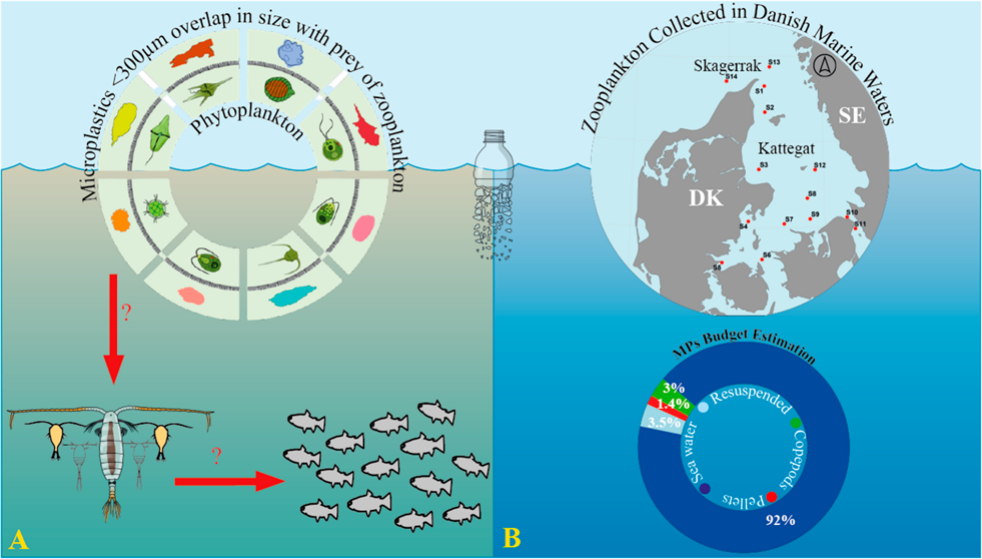

Microplastics (MPs) overlap in size with phytoplankton and can be ingested by zooplankton, transferring them to higher trophic levels. Copepods are the most abundant metazoans among zooplankton and the main link between primary producers and higher trophic levels. Ingestion of MPs has been investigated in the laboratory, but we still know little about the ingestion of MPs by zooplankton in the natural environment. In this study, we determined the concentration and characteristics of MPs down to 10 μm in zooplankton samples, sorted calanoid copepods, and fecal pellets collected in the Kattegat/Skagerrak Sea (Denmark). We found a median concentration of 1.7 × 10^–3^ MPs ind^–1^ in the zooplankton samples, 2.9 × 10^–3^ MPs ind^–1^ in the sorted-copepods, and 3 × 10^–3^ MPs per fecal pellet. Most MPs in the zooplankton samples and fecal pellets were fragments smaller than 100 μm, whereas fibers dominated in the sorted copepods. Based on the collected data, we estimated a MP budget for the surface layer (0–18 m), where copepods contained only 3% of the MPs in the water, while 5% of the MPs were packed in fecal pellets. However, the number of MPs exported daily to the pycnocline via fecal pellets was estimated to be 1.4% of the total MPs in the surface layer. Our results indicate that zooplankton are an entry point of small MPs in the food web, but the number of MPs in zooplankton and their fecal pellets was low compared with the number of MPs found in the water column and the occurrence and/or ingestion of MPs reported for nekton. This suggests a low risk of MP transferring to higher trophic levels through zooplankton and a quantitatively low, but ecologically relevant, contribution of fecal pellets to the vertical exportation of MPs in the ocean.

## Abstract 2

We found a low concentration of microplastics in zooplankton and their fecal pellets, suggesting a low risk of ingestion and biotransfer of microplastics via zooplankton.

## 1 Introduction

Microplastics (MPs, 1 μm–5 mm^1^) are ubiquitous pollutants in aquatic environments, and their potential environmental impacts are a major global concern.^2−6^ Recently, there has been increasing interest in small-size MPs fractions (<300 μm)^7^ since they overlap in size with the natural prey of zooplankton.^8−10^ Recent studies show that MPs < 300 μm are the dominant size fraction in marine waters,^11−13^ increasing the risk of MPs entering marine food webs via zooplankton ingestion. Thus, given their high abundance and key position in marine ecosystems, zooplankton could be an entry point for MPs into the food web.

Among zooplankton, copepods dominate the metazoan biomass in the ocean.^14^ These crustaceans are key players in marine food webs since they constitute a main link between phytoplankton and higher trophic levels.^15−17^ Numerous laboratory investigations have shown that copepods ingest more MPs as exposure concentrations increase.^18−21^ Laboratory studies have shown that ingestion of MPs may cause adverse effects on copepods, such as reduced grazing, reproduction, and egestion,^22−24^ or noneffects,^25^ depending on the species,^26^ life stages,^27^ and, particularly, on the concentration and characteristics of the MPs.^28^ However, the concentrations of MPs used in laboratory studies are extremely high, several orders of magnitude higher than what is found in the natural environment,^29^ and the observed high ingestion of MPs in these laboratory experiments could be an artifact. Field studies on MP ingestion in zooplankton are still limited, and the results are disparate, from no evidence/low ingestion of MPs^30,31^ to a high occurrence and ingestion of MPs in zooplankton.^32,33^ However, in some studies, the size of ingested MPs was outside the size range of natural prey and even larger than the mouth opening of the copepods (e.g., Zheng et al., 2020^34^), suggesting entanglement or contamination rather than ingestion. Thus, the risk of ingesting of MPs by zooplankton in the sea is still unclear, and more research is needed to quantify the ingestion of small-size MPs in zooplankton.

Planktonic copepods contribute to global biochemical cycles: for instance, copepod fecal pellets are exported to the deep ocean, contributing to carbon sequestration as part of the biological carbon pump.^35^ MPs are packed in fecal pellets after being ingested by copepods or other zooplankton.^25^ In the context of plastic pollution, zooplankton fecal pellets could play a role in the vertical distribution of MPs and associated additives. However, there are only a few field studies on the occurrence and concentration of MPs in zooplankton fecal pellets.^31,36^ Therefore, ingestion of MPs by zooplankton deserves special attention since it can be an entry point and vector of MPs in the marine food webs and affect the vertical exportation of MPs via fecal pellets.

In this study, we aim to evaluate the significance of zooplankton as a potential pathway for the entry of MPs into marine food webs. To achieve this, we investigated the concentration and characteristics of small MPs (<300 μm) within zooplankton communities collected at different depths of 14 stations at Kattegat Strait and Skagerrak, Denmark. The Kattegat and Skagerrak along with the Great Belt, Little Belt, and Øresund (The Sound) serve as the primary connecting channels between the Baltic Sea and the North Sea. We also examined sorted copepod samples and zooplankton fecal pellets. Our group investigated and published the abundance of MPs down to 10 μm in surface waters^13^ at the same sampling stations that were examined in this study. Our findings contribute to evaluating the risk of MPs uptake by zooplankton, the potential transfer of MPs to higher trophic levels, and the role of zooplankton in the vertical exportation of MPs through fecal pellets.

## 2 Materials and Methods

### 2.1 Collection of Zooplankton Samples

**Figure 1:**
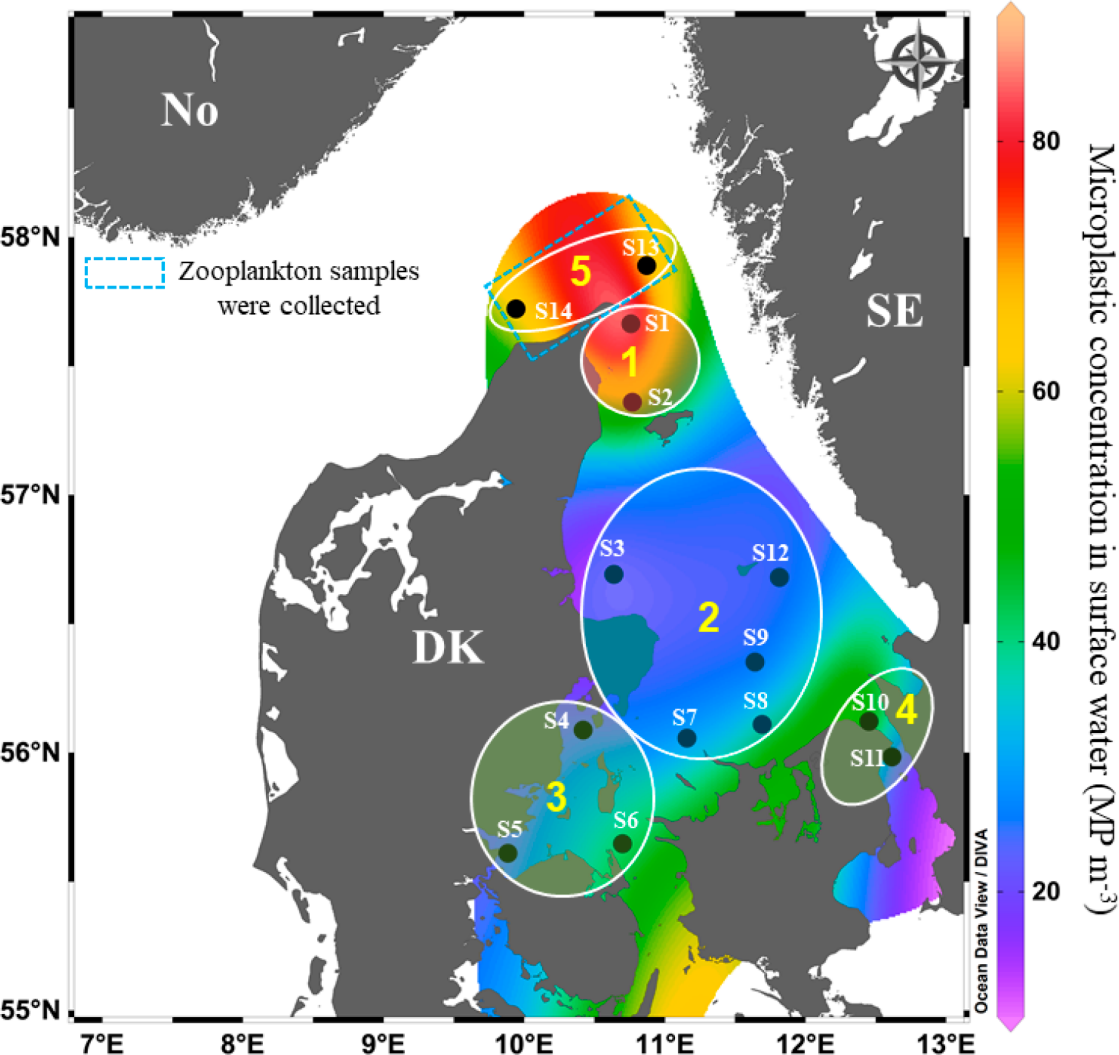
Locations/stations (S1–S14) in the Kattegat/Skagerrak where the zooplankton samples were collected. The discontinuous square indicates the stations (S13 and S14) where zooplankton samples from different depths were collected. Sorted copepod samples from different stations were pooled in five samples, corresponding to different zones in the Kattegat/Skagerrak (1–5) (encircled stations). The concentration and distribution of MPs in surface water were adopted from Gunaalan et al., 2023.^13^

Zooplankton samples were collected from 14 stations in the Kattegat and Skagerrak (Figure 1) during a cruise on board R/V DANA (DTU Aqua) from 20th October to 1st November 2020. The general hydrography and sampling locations of the studied area is described in Gunaalan et al., 2023.^13^ A Multi-Net (MOCNESS; Hydro-Bios, Kiel, Germany) consisting of five nets (mesh size 335 μm) attached to a stainless-steel frame opening (0.25 m^2^) was used to collect the zooplankton from different water layers (Figure S1a). The multinet was towed obliquely, and the net bags closed at selected depths strata: surface water (above the pycnocline), midwaters (pycnocline), and deep water (below the pycnocline) in all stations except for the deepest station (St. 13), where five different depths were sampled. The sampling depths of the stations were determined according to profiles obtained in each station with a CTD (Sea-Bird SBE 9). At each station, the cod ends containing the zooplankton samples were kept separate in closed metal buckets until processing in the onboard laboratory.

The zooplankton samples collected with the Multinet (335 μm) from the different depth strata were concentrated using a 300 μm metal sieve and subsequently divided into two subsamples onboard using a Folsom’s plankton splitter. One subsample of 250 mL was fixed with buffered formaldehyde (4%) for further analyses of the abundance, composition, and vertical distribution of zooplankton.

### 2.2 Sorted Copepods from Surface Water Samples for MPs Analysis

At all stations, copepods were sorted from zooplankton subsamples for estimating MP ingestion. In most of the cases, it was a mix of calanoids, except in some stations where the calanoid community was dominated by *Acartia tonsa* (Figure S2). The most abundant calanoid copepods in each station were randomly sorted. In order to sort the copepods from the zooplankton subsamples, the subsamples were concentrated in 100 mL using a 300 μm metal sieve and then precise aliquots of several mL were placed in glass Petri dishes examined under a stereomicroscope. We specifically chose to sort from the surface the water samples as zooplankton tended to be more prevalent in surface water at most stations. Approximately 200 calanoid copepods per station were identified and sorted from each surface water sample. The copepods were rinsed three times by sequential transferring of individual copepods to glass Petri dishes with 0.2 μm filtered seawater (FSW). The separated copepods were placed into a muffled 20 mL glass vial with 5% sodium dodecyl sulfate (SDS; diluted with Milli-Q water) to ensure the solubilizing of samples and denature the proteins in the samples. The copepod samples were pooled based on the MPs concentration in the surface water at Kattegat/Skagerrak^13^ (Figure 1) in order to increase the MPs detection sensitivity. Hereinafter these samples are referred to as “sorted-copepod samples”.

### 2.3 Zooplankton Community Samples from Different Depths for MPs Analysis

Two deeper study sites, St. 13 and St. 14, were investigated for determining the concentration and characteristics of MPs in the entire zooplankton samples collected from different depths (Table 1). Two samplings were conducted at St. 13: one in the morning (St. 13a) and one at night (St. 13b). The sampling at station 14 was conducted the next day in the morning. On board, the content of the cod end was concentrated on a 300 μm metal sieve (concentrated sample volume = 100 mL). An aliquot of 10 mL of the concentrated sample was taken using a glass tube attached to a 10 mL automatic pipet and then fixed with buffered formaldehyde (4%) to determine the concentration and composition of zooplankton. The rest of the concentrated zooplankton sample (90 mL) was placed in a glass jar with 100 mL of 5% SDS to start the sample preparation for analyses of MPs. From now on, they are referred to as “zooplankton samples.”

**Table 1:** Summary of the Concentration of MPs Found in Zooplankton Samples, Sorted-Copepods, and Fecal Pellet Samples

Measurement	Sample No.	Depth range (m)	Sample volume (m^3^)	Number of Individuals or fecal pellets per sample	Number of MPs before blank correction	Number of MPs after blank correction	Conc. MPs in filtered water through multinet (MPs m^–3^)	Conc. MPs (MPs ind^–1^)	Mass estimates of MPs before blank correction (μg)	Mass estimates of MPs after blank correction (μg)	Conc. Mass estimates of MPs in filtered water through multinet (μg m^–3^)	Conc. Mass estimates of MPs (μg ind^–1^)
Zooplankton Samples	St.13 Sampling ‘a’	0–10	135	8680	25.1	17.2	0.13	0.0020	3.540	3.059	0.02266	0.00035
		10–30	89	9009	24.8	16.8	0.19	0.0019	15.970	15.484	0.17398	0.00172
		30–50	122	12060	24.4	16.5	0.14	0.0014	1.060	0.575	0.00471	0.00005
		50–70	103	7578	22.5	14.6	0.14	0.0019	22.800	22.320	0.21670	0.00295
		70–90	95	8136	94.5	86.6	0.91	0.0106	137.540	137.056	1.44269	0.01685
	St.13 Sampling ‘b’	0–10	117	2810	42.2	34.3	0.29	0.0122	10.220	9.735	0.08320	0.00346
		10–30	109	13790	4.4	0.0	0.00	0.0000	0.240	0.000	0.00000	0.00000
		30–50	109	14350	20.0	12.1	0.11	0.0008	0.150	0.134	0.00123	0.00001
		50–70	109	4010	8.9	1.0	0.01	0.0002	0.190	0.025	0.00023	0.00001
		70–90	103	1380	8.9	1.0	0.01	0.0007	0.040	0.036	0.00035	0.00003
	St.14	0–40	119	8352	117.0	109.1	0.92	0.0131	94.860	94.377	0.79309	0.01130
		40–60	104	12537	28.7	20.8	0.20	0.0017	7.987	7.505	0.07216	0.00060
		60–80	62	6561	6.8	0.0	0.00	0.0000	0.263	0.000	0.00000	0.00000
Sorted-copepod samples	Zone I	Surface		800	17.1	2.3	N/A	0.0029	10.220	8.979	N/A	0.01122
	Zone II	Surface		1137	0	0.0		0.0000	0.000	0.000		0.00000
	Zone III	Surface		800	30.0	10.8		0.0135	2.790	1.069		0.00134
	Zone IV	Surface		451	42.9	28.0		0.0621	81.230	79.986		0.17735
	Zone V	Surface		800	5.5	0.0		0.0000	0.810	0.000		0.00000
Fecal pellets	Zone I	10–25 m		600	39.5	24.6	N/A	0.0410	1.390	0.144	N/A	0.00024
	Zone II			780	17.4	0.0		0.0000	0.480	0.000		0.00000
	Zone III			619	16.4	0.0		0.0000	0.160	0.000		0.00000
	Zone IV			400	18.9	4.1		0.0102	0.500	0.481		0.00120
	Zone V			400	15.0	0.1		0.0003	27.760	0.257		0.00064

### 2.4 Fecal Pellet Samples

Zooplankton fecal pellet samples were collected at all stations, except for station 8 where the samples were lost. The fecal pellet samples were collected using a metal floating sediment trap (KC Denmark A/S) consisting of two parallel cylinder tubes with a diameter and length of 80 and 450 mm, respectively (Figure S1 a) deployed at the beginning of the pycnocline (10–25 m) for 6–8 h. The contents of the sediment traps were concentrated with a 22 μm metal sieve, and the fecal pellets were identified and sorted under a stereomicroscope. 100–200 fecal pellets were separated at each station, rinsed three times by sequentially transferring individual pellets to a glass Petri dish with FSW, and placed into a 20 mL glass vial with 5% SDS. These fecal pellet samples were pooled like the sorted copepods (Figure 1). Additionally, concentrations of zooplankton fecal pellets in the surface waters of stations 1–12 were estimated from samples taken at 5 m by a Niskin bottle (20 L) mounted on the CTD Rosette in order to calculate the sinking velocity of the fecal pellets.

### 2.5 Preparation of Samples for MPs Analysis

Once in the laboratory, zooplankton samples were prepared for analyses of MPs using a slightly modified protocol of the enzymatic-oxidative process described in Löder et al., 2017^37^ (Figure S1 b). Initially, the samples were placed into a beaker with 5% SDS for 24 h at 50 °C before being filtered using 10 μm steel filters (Ø = 47 mm). Then, they were incubated at 50 °C for 48 h in protease (Sigma, protease from *Bacillus sp*.), with the successive addition of 30% H_2_O_2_ and kept at room temperature for another 48 h. After filtration using 10 μm steel filters, Chitinase (ASA Spezialenzyme, GmbH) was introduced and maintained in a 37 °C water bath for 5 days. An additional dose of approximately 30% H_2_O_2_ was added, and the samples were allowed for another 48 h of incubation at room temperature. The samples were then filtered again using 10 μm steel filters. Next, MPs were separated using sodium polytungstate (SPT, 1.7 g cm^–3^), and the floating fraction was separated, briefly sonicated, and washed with 50% ultrapure ethanol. Finally, all liquid was gradually transferred to 10 mL muffled glass vials and evaporated in a water bath at 50 °C using a stream of nitrogen (Biotage, TurboVap).

### 2.6 MPs Detection and Data Analysis

Ultrapure ethanol (3 mL) was added to the vial with the evaporated sample and homogenized using a vortex. Using a disposable capillary glass pipet (microclassic, Brand GmbH, Germany), an aliquot equal to about 50% of the sample was placed onto zinc selenide (ZnSe) infrared windows (Crystran, UK, Ø = 13 mm, *t* = 2 mm) in a compression cell (PIKE Technologies, Fitchburg, WI, USA). The deposited samples were dried at 50 °C. Finally, an integrated system consisting of an FTIR microscope (Cary 620) with a focal plane array (FPA) detector (128 × 128) and a FTIR spectrometer (Cary 670, Agilent Technologies, Santa Clara, CA, USA) was used to scan the whole active surface of the ZnSe window for co-adding 30 scans for each tile in transmission mode (Spectral range = 3750–850 cm^–1^; Resolution = 8 cm^–1^). The freeware siMPle (https://simple-plastics.eu/) was utilized to perform an automated analysis of large spectral data set obtained from FPA-μFTIR-imaging.^38^ The software performs a Pearson correlation between each sample spectrum and reference spectra contained in a custom-built database, and it provides the chemical identification of the sample’s particles, as well as information on their size, volume, and mass estimates^39^ (Figure S1c). All MP particles were identified as fibers or fragments based on the ratio between the length and width, which defines the fiber as an object with a length-to-width ratio greater than 3, and the fragments were defined as objects with a length-to-width ratio ≤3.^40,41^

### 2.7 Quality Control of MPs Sampling and Sample Preparation

The samples were processed by following strict quality control and assurance protocols. The processing was carried out under a laminar flow hood, and cotton lab coats were always worn. The use of plastic-containing materials and equipment was minimized during sampling and sample analysis, and any unavoidable plastic materials were identified and excluded from MPs quantification. In addition, we collected samples from all possible cross-contamination points, including the ship’s paints. The matching paint particles in the samples were excluded. All materials were rinsed with Milli-Q water, muffled at 500 °C, and wrapped in aluminum foil until use. All utilized chemical solutions were filtered over 0.7 μm GF/F filters. The study also examined and quantified the potential for field and procedural cross-contamination of MPs through analysis of “air blanks” from the ship, water blanks from the ship’s workstation, and procedural blanks from the laboratory. The “air blanks” were collected by opening a muffled Petri dish every time the sample was transferred from multinet to the glass bottle containers. Another Petri dish was placed next to the ship’s workstation, and throughout the whole cruise, it was left exposed only during sample analysis and preparation. While the copepods and fecal pellets were sorting, water blanks from the ship’s workstation were also collected. Furthermore, procedural blanks were obtained, including all lab reagents and materials without a sample.

### 2.8 MP Budget in the Water Column and Export of MPs via Fecal Pellets

A budget of MPs available to zooplankton in the water column was estimated based on the estimated mean concentration of MPs (MPs m^–3^) found in surface waters during the same survey by Gunaalan et al., 2023^13^ and the median concentration of MPs in copepods (MPs ind^–1^) and fecal pellets (MPs pellet^–1^). The number of MPs in copepods was estimated by multiplying the median concentration of MPs per copepod by the abundance of copepods larger than 300 μm (the fraction of the copepod community able to ingest MP > 10 μm). Furthermore, based on the copepod mouth size^42,43^ it was assumed that the copepods ingest only MPs smaller than 100 μm. The fecal pellets’ sedimentation rate and sinking velocity were calculated using the equation from Knap et al., 1996^44^ and Kiørboe et al., 1994,^45^ respectively:
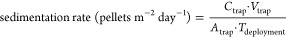
1where *C*_trap_ is the concentration of fecal pellets in the trap (pellets m^–3^), *V*_trap_ is the volume of the sediment trap (m^3^), *A*_trap_ is the surface area of the sediment trap (m^2^), and *T*_deployment_ is the deployment time (day);
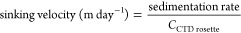
2where the sedimentation rate is the result from eq 1 and the *C*_CTD rosette_ is the fecal pellet concentration (pellets m^–3^) in the first 5 m of the surface water column, measured from water samples from the Niskin bottles. The CTD profiles were taken just prior to the placement of the sediment traps.

### 2.9 Data Handling and Statistical Analysis

#### 2.9.1 Blank Correction

A blank correction for samples was done based on both the field (“air blank” and “water blank”) and procedural blanks. The “air blank” correction was performed based on the handling time of the samples at the ship and the opening area of the glass container (Table S1). For instance, the handling time for zooplankton samples was approximately 1 h per sample, while copepod and fecal pellet sorting took around 8 h per sample. Eventually, the “air blank” and water blanks from the ship’s workstation, as well as procedural blanks, were used to correct the measured MPs from the samples (Table S1).

#### 2.9.2 Statistical Analysis

Descriptive statistics were used to analyze the blank-corrected data based on the abundance, polymer type, size, and estimated mass of MPs. The Kruskal–Wallis test was applied to compare the size of MPs in surface water,^13^ zooplankton samples, sorted-copepod samples, and fecal pellets followed by pairwise comparisons using Dunn’s test. In order to assess differences in the polymer compositions among the types of samples, Fisher’s exact test was conducted. The significance level for all tests was set at α = 0.05. The statistical program R (version 4.2.1, R Core Team (2022)) was used to analyze all of the data.

## 3 Results

### 3.1 Blank Correction

We found that zooplankton samples were contaminated by 1.8 MPs per sample. On the other hand, sorted samples (copepods and fecal pellets) were corrected by 4.4 MPs per sample. A median of 6.2 MPs per sample of water blanks from the ship’s workstation and procedural blank was corrected from all of the samples (Table S1). Moreover, the results were corrected for contamination by subtracting the contribution of every single polymer found in the blanks. When this led to negative values in the samples, these were set to zero. When examining the “air blanks”, polyester (77%) was the prevalent polymer followed by polyamide (10%), polyacrylonitrile fiber (5%), and acrylic paint (3%). In contrast, polyester (76%) was also the dominant polymer in the water blanks from ship’s workstation and procedural blanks followed by 12% polypropylene, and 6% of acrylic paint was also recorded in these blanks. After implementing the required blank correction, we noticed substantial changes in the average MP composition by number and mass in sorted copepod samples and fecal pellets that underwent extended processing for separation during sampling (Figure S3).

### 3.2 Concentration of MPs in Zooplankton Samples, Sorted Copepods, and Fecal Pellets

**Figure 2:**
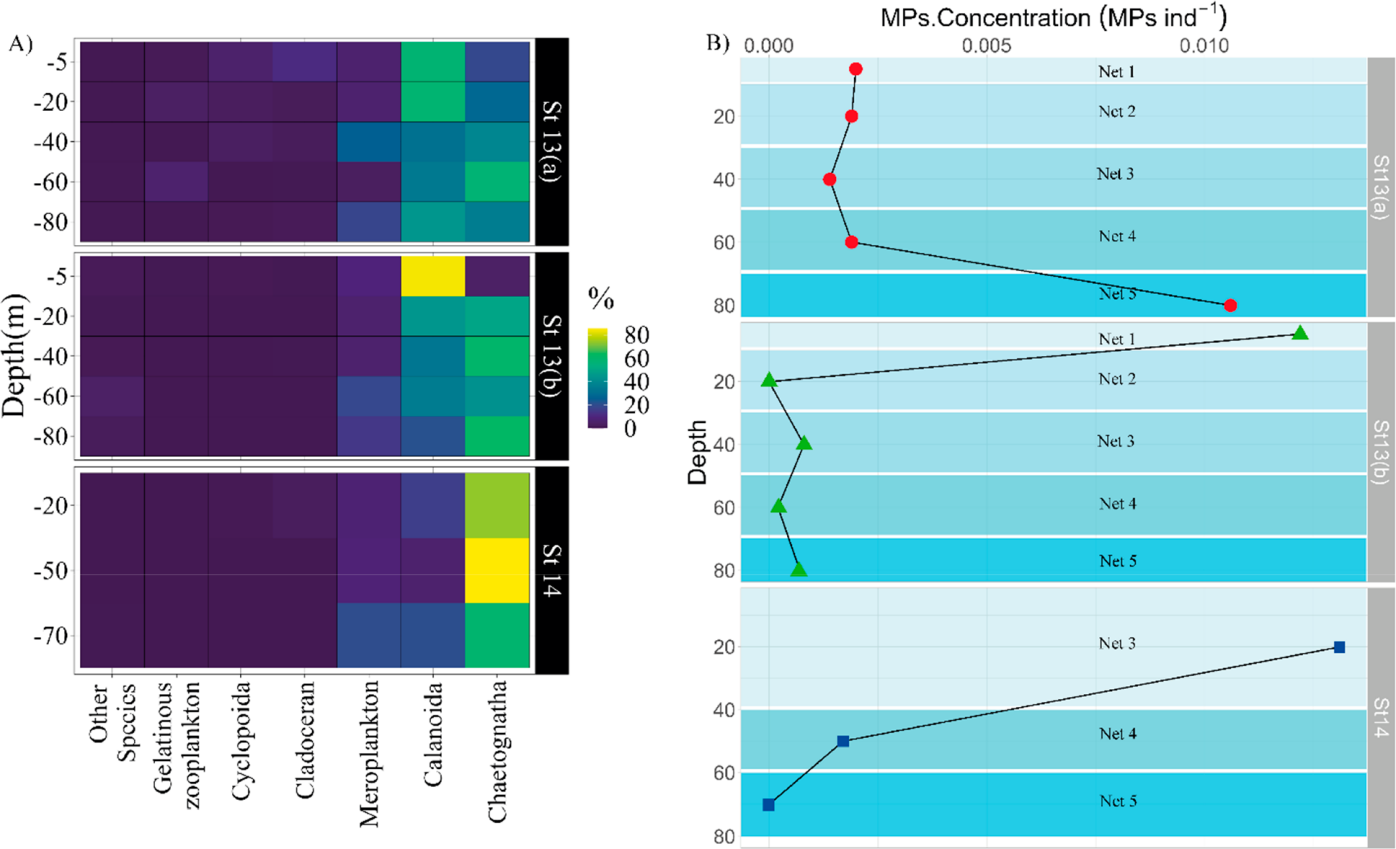
Vertical distribution at stations 13 (13a: day; 13b: night) and 14 of (A) contribution of zooplankton taxa to total community abundance and (B) MPs concentration (MPs ind^–1^) in zooplankton samples.

Overall during our study, cyclopoid (38 ± 15%) and calanoid (25 ± 13%) copepods and meroplankton (14 ± 18%) dominated zooplankton abundances, followed by harpacticoid copepods (5 ± 6%) (Figure S4). At stations 13 and 14, zooplankton were more diversified than at the other stations and were dominated by chaetognaths (47 ± 22%), calanoid copepods (37 ± 21%), and meroplankton (11 ± 7%) (Figure 2A). The concentration of MPs in the samples from stations 13 and 14 ranged from 0 to 0.92 MPs m^–3^ (median = 0.14 MPs m^–3^, mean: 0.23 ± 0.31 MPs m^–3^) (Table 1). Considering the number of individuals, the concentration of MPs in the zooplankton samples ranged from 0 to 0.0131 MPs ind^–1^ and the median number of MPs per individual in the zooplankton samples was 0.0017 MP ind^–1^ (0.0031 ± 0.0049) (Figure 2B; Table 1). There were no significant differences in the concentrations of MPs ind^–1^ between stations (13 vs 14) or sampling time in St. 13. The maximum concentration of MP ind^–1^ was observed in deep water (70–90m) samples collected in St. 13a, surface (0–10m) samples collected in St. 13b, and St. 14 at 20 m depth (Figure 2B).

The sorted copepod samples were dominated by calanoids where *Acartia* sp. prevailed in most of the stations (Figure S2), and the median concentration was 0.0029 MPs ind^–1^ (mean ± SD: 0.0157 ± 0.0265). We did not find MPs in the sorted copepod samples from zone II (offshore/central Kattegat stations) or zone V (Skagerrak) (Table 1; Figure 1). The concentration of MPs in the zooplankton fecal pellet samples collected from the sediment trap was low with a median concentration of 0.0003 MPs pellet^–1^ (mean ± SD, 0.0103 ± 0.0177) and zero MPs pellet^–1^ in zones II and III (Table 1). On the other hand, there was a high degree of variability in the concentration of mass estimates of MPs among the samples. The zooplankton samples collected from stations 13 and 14 exhibited an average of 0.42 ± 0.21 μg m^–3^ (with a median of 0.02 μg m^–3^). Similarly, when considering the number of individuals, the concentration of MP mass estimates in the zooplankton samples was 0.0028 ± 0.0051 μg ind^–1^(median: 0.0004 μg ind^–1^). The sorted-copepod samples had an average concentration of 0.04 ± 0.08 μg ind^–1^ (median: 0.001 μg ind^–1^), while the fecal pellets showed a mean concentration of 0.0004 ± 0.0005 μg ind^–1^ (with a median of 0.0002 μg ind^–1^) (Table 1).

### 3.3 Plastic Particle Shape (Fragment vs Fiber) and Polymer Composition

Most of the MPs were fragments in the zooplankton samples (59%) and fecal pellets (76%), whereas they were fibers in the sorted-copepod samples (55%) (Figure S5). In all categories, polyester and polypropylene were the dominant polymers. Most MPs were identified as polyester fibers in sorted-copepods (83%) and in zooplankton samples (61%) (Figure S5).

**Figure 3:**
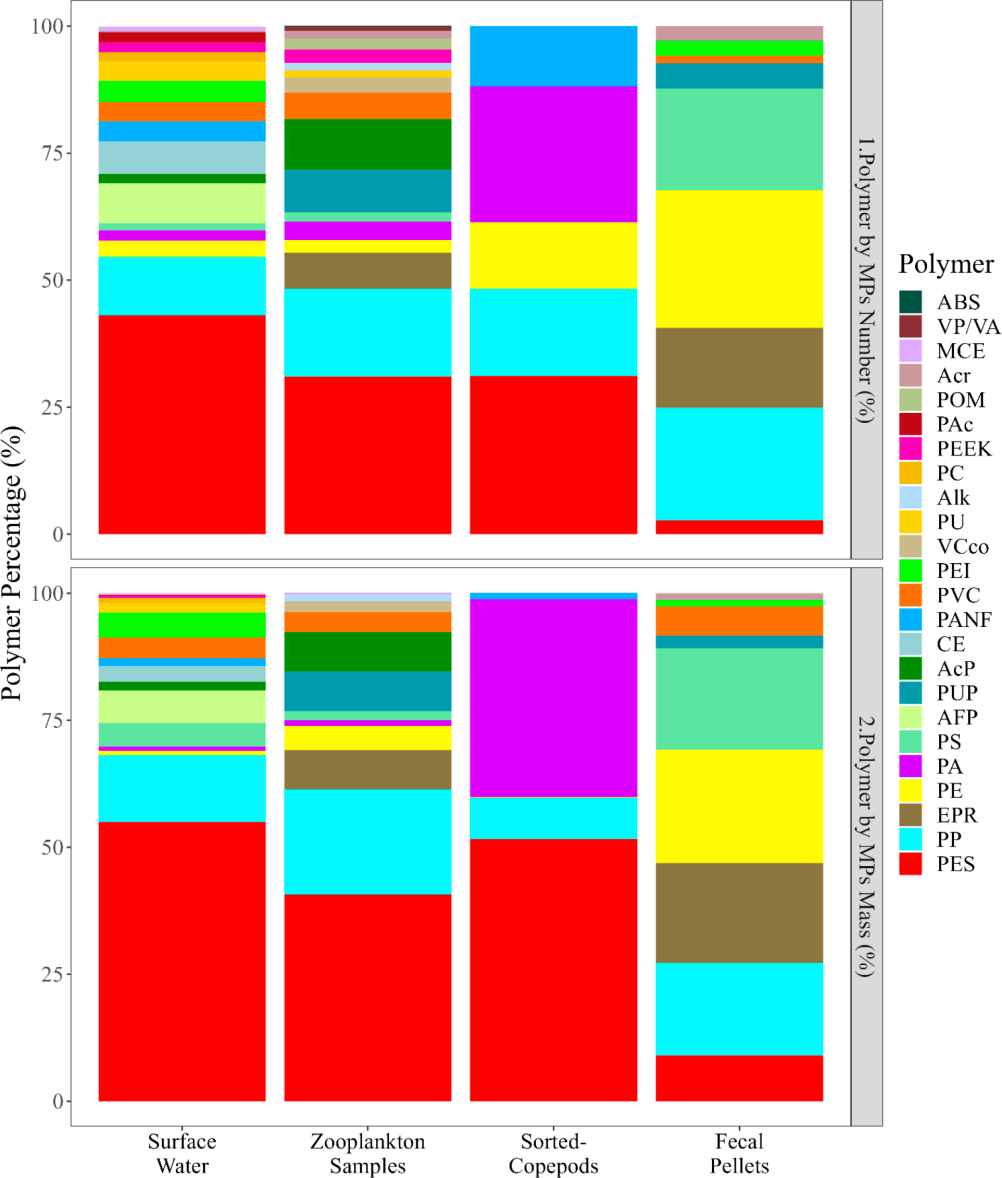
Average percentage of polymer composition in surface water,^13^ zooplankton samples, sorted-copepod samples, and fecal pellets. The upper panel shows the percentage of polymer composition based on numbers and the lower panel illustrates the percentage of polymer composition based on mass estimates of the MPs. (ABS: Acrylonitrile butadiene styrene, AcP: Acrylic paint, Acr: Acrylic, AFP: Antifouling paint, Alk: Alkyd, CE: Cellulose ester, EPR: Epoxy phenoxy resin, MCE: Modified cellulose ester, PA: Polyamide, PANF: PAN acrylic fiber, PC: Polycarbonate, PE: Polyethylene, PEEK: Polyether ether ketone, PEI: Polyethylenimine, PAc: Polyacrylamide, PES: Polyester, POM: Polyoxymethylene, PP: Polypropylene, PS: Polystyrene, PU: Polyurethane, PUP; PU paint, PVC: Polyvinylchloride, PVDF: Polyvinylidene fluoride, VCco: Vinyl chloride copolymer, VP/VA: Polyvinylpyrrolidone/Vinyl Acetate)

The polymer composition and proportion in terms of numbers and masses of MPs varied considerably among the samples (Figure 3). The samples contained a total of 24 different types of polymers, with only five types identified in the sorted copepods such as polyester (31%), polypropylene (17%), polyamide (27%), polyethylene (13%), and polyacrylonitrile fiber (12%). Many polymers found in zooplankton and fecal pellets were also present in the surface water,^13^ but their numbers and mass differed. Fisher’s exact test confirmed a relationship between the polymer composition among the samples (*p* = 0.0004).

### 3.4 Size of MPs

**Figure 4:**
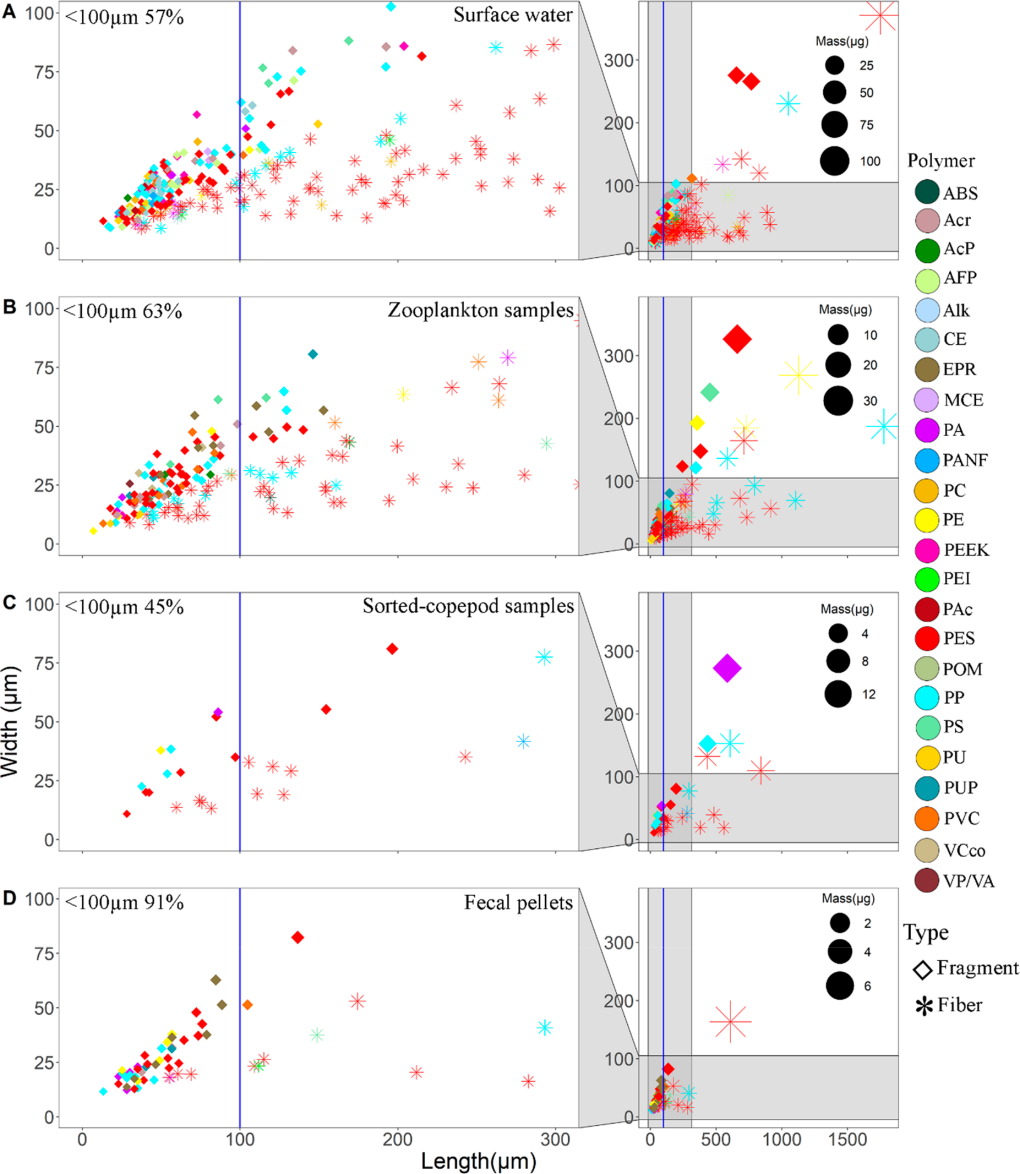
Distribution of particle size (length and width) and mass of the MPs in the surface water^13^ (A), zooplankton samples (B), sorted copepods (C), and fecal pellets (D) in the study area. The right panel displays the distribution of size, shape, mass, and polymer characteristics of all MPs, while the left panel provides a zoomed-in view specifically showing MPs that were less than 300 μm in length. The section to the left of the blue vertical line illustrates the MPs particles that were less than 100 μm. This particular size range suggests a potential for ingestion by planktonic copepods. Additionally, the percentage of the length below 100 μm is displayed on the top left corner. (Acronyms are as in Figure 3.)

Considering the total number of MPs found in all our samples, 88 ± 8% (mean ± SD) was smaller than 300 μm, and 62 ± 13% was smaller than 100 μm. The fecal pellets exhibited the highest percent of MPs < 100 μm, accounting for 91%, whereas the sorted copepods had the lowest proportion at 45% (Figure 4). Additionally, the majority of MP fragment’s lengths were found below 100 μm (Figure 4). There were significant differences (*p* < 0.05) in the length of MPs among the samples, except for surface water and zooplankton samples (*p* > 0.05) (Figure S6). The length–frequency peak of MP fragments was less than 100 μm in all samples. In addition, there was a substantial variation in the length of MP fibers compared to the length of the fragments (Table S2).

Concerning the mass of the polymers, polyester and polypropylene accounted for around 50% of the total polymer composition in all samples except fecal pellets (Figure 3). The total mass of the MPs was highly variable in surface water, zooplankton samples, sorted-copepod samples, and fecal pellets, where the estimations were 210.2, 131.2, 33.2, and 8.9 μg, respectively. Overall, the mass of the MP particle types also considerably varied among the samples (Table S2).

### 3.5 MP Budget in the Water Column

The average concentration of MPs was 39 MPs m^–3^ in surface waters.^13^ Approximately 3% of MPs were in copepods, and 5% of MPs were inside the fecal pellets (Table S3). Assuming that copepods, fecal pellets, and MPs in the water column were uniformly distributed above the pycnocline, we established a budget for MPs assuming a mean depth of the pycnocline of 18 m. The mean sedimentation rate was 46383 pellets m^–2^ day^–1^ with a sinking velocity of 11.1 m per day (*n* = 11, Table S4). We estimated that 154494 fecal pellets m^–2^ were present in the surface layer and 95271 pellets m^–2^ could be potentially exported to the pycnocline daily. However, only 46383 pellets m^–2^ were collected in the sediment trap, suggesting that significant pellet degradation takes place in the surface layer. From the 5% of MPs present in fecal pellets in surface waters, only approximately one-third of the MPs were exported across the pycnocline, while the remaining MPs were likely reintroduced to the water column through the degradation of fecal pellets (Figure S7).

## 4 Discussion

### 4.1 Contamination/Quality Control/Blank Corrections

Since many materials/clothes are made of synthetic polymers, contamination of samples during laboratory processing should be minimized. Blank corrections are especially crucial for environmental samples that contain low concentrations of MPs and require long laboratory processing times. Our study found that handling samples in the laboratory, such as for sorting copepods and fecal pellets for several hours under the microscope, increased sample contamination, particularly with polyester (Figure S3), which is typically found in synthetic clothing materials. Our study clearly demonstrated that, before blank correction, the concentration of MPs in zooplankton samples was lower than in sorted copepods and fecal pellets, indicating that MP environmental contamination is high in long-time processed samples (Figure S3). This correction decreased the estimated concentration by approximately 2.5 orders of magnitude. After correcting the data with the blanks, the concentration of MPs in the sorted samples was like those found in short-time processed samples, i.e., zooplankton samples. Therefore, establishing quality controls and proper corrections of contamination is critical to avoid an overestimation of the abundance of MP in environmental samples, such as in hand-sorted zooplankton and fecal pellet samples.

### 4.2 Concentration of MPs in Zooplankton and Fecal pellets

The zooplankton samples were collected using a 335 μm mesh net, and MPs bigger than 335 μm can be entangled with the zooplankton or otherwise be outside of bodies. Thus, we cannot assume that all MPs larger than 335 μm are ingested. Still, MPs > 300 μm represented a minor fraction of the total MPs and they are not likely ingested by copepods since they are larger than their mouth size or outside of the size spectra of the other dominant zooplankton in our samples. Regarding the small size MPs (<300 μm), since the samples were collected with a 335 μm mesh and additionally filtered and rinsed on a 300 μm metal sieve, the presence of free MPs >300 μm in our samples was minimized. However, the concentration of MPs found in our zooplankton samples was very low (see Table 1), even lower than in sorted copepods. Additionally, the fact that some of the detected MPs could be external to zooplankton helps to support our conclusion about the low ingestion of MPs in zooplankton.

Field studies on the ingestion of MPs by zooplankton report quantities of ingested particles that vary by several orders of magnitude depending on the location, zooplankton species/groups, methods used for sample collection, sample treatment, and MP detection methods (e.g.^28,30,34,46−50^). One important reason for this variation is the diversity of analytical methods applied for the identification of MPs. While most methods can quantify large MPs (above 500 μm) with good accuracy, the accuracy for all methods decreases when particles get smaller. However, some methods are more suited to detect small MPs than others, and the choice of the analytical approach is, therefore, a major factor in the number of MPs found during analysis. We observed that the concentration of MPs in zooplankton was much lower (<0.002 MPs ind^–1^) than in other studies, e.g. Md Amin et al., 2020,^48^ and Aytan et al., 2022^31^ (Table S5). Additionally, our results show no or a low occurrence of MPs in copepods. However, other studies found a very high occurrence of MPs in copepods, up to 2 orders of magnitude higher than in our study for similar-sized zooplankton species (e.g., *Acartia tonsa*).^33^ Furthermore, the estimated MP budget (Figure S7) shows that the percentage of MPs in copepods is very low (3% of the total MPs in the water column are found inside the copepods).

Encounter rates between MPs and zooplankton are affected by the MP concentrations in the water column (zooplankton/MPs ratio). Botterell et al., 2022^49^ found high ingestion of MPs in zooplankton from the Arctic Fram Strait, where the concentration of MPs in surface water was very high (0–18500 MPs m^–3^). The concentration of MPs in surface waters^13^ in our study area, 11–87 MPs m^–3^, is lower than in the Arctic Fram Strait, reducing the risk of zooplankton encountering MPs. Even so, no ingestion of MPs by zooplankton has been reported in highly polluted environments like harbors.^30^

Zooplankton are composed of very diversified assemblages of organisms with different sizes and foraging behaviors. As expected, we found a higher diversity of zooplankton in stations 13 and 14, close to/at Skagerrak due to the more oceanic characteristics, including higher salinity and deeper waters.^51^ Previous field studies indicate that copepods contain fewer MPs than other groups like medusae^34^ and amphipods.^49^ This difference among zooplankton groups can be related to different feeding strategies. Planktonic copepods can discriminate MPs from similar-sized prey.^52,53^ Interestingly Xu et al., 2022^53^ proved that feeding-current generating copepods rejected 80% of the MPs by postcapture, and the rejection rates were unaffected by the type of polymer, shape, presence of biofilms, or the sorbed pollutant investigated in that study. In the case of ambush zooplankton (e.g., cyclopoid copepods *Oithona* sp.), clearance rates on nonmotile prey or particles like MPs are very low,^54,55^ reducing the risk of MPs ingestion. Besides copepods, chaetognaths were a dominant component of the zooplankton community at stations 13 and 14 (Figure 2A). Chaetognaths are rheotactic predators that feed on copepods; therefore, the direct ingestion of nonmotile MPs is unlikely. However, it is also probable for chaetognaths to indirectly consume MPs through the ingestion of copepods that may have already ingested MPs. The risk of ingestion or entanglement can be higher for some zooplankton groups that use other foraging mechanisms like mucus filter structures (some gelatinous zooplankton and larvaceans),^36^ visual predation (fish larvae), or in benthic copepods that feed on marine-snow/aggregates (e.g., *Oncaea* sp.).

If copepods ingest MPs, the particles are rapidly egested through fecal pellets; therefore, the residence time of MPs in the copepod is short. However, it is worth noting the egestion rates could differ among the species up to 2–168 h.^8,26^ Aytan et al., 2022^31^ found 4 MPs after examining 351 field-collected fecal pellets (0.011 MPs pellet^–1^), whereas we found lower concentrations of MPs in our samples (0.0003 MPs pellet^–1^, *n* = ca. 2800 fecal pellets).

### 4.3 Characteristics of Ingested MPs

The bioavailability and ingestion of MPs by zooplankton also depend on the characteristics of the MPs, such as size, shape, polymer type, and presence of biofilms.^26,56−58^ Particle size is crucial for evaluating the risk of MP ingestion by zooplankton. Small-size MPs (<300 μm) overlap in size with the common prey of zooplankton (e.g., phytoplankton, protozoans). Still, zooplankton shows different size selectivity spectra and optimal predator-to-prey ratio (maximum clearance rates) depending on taxonomy.^59^ Sensorial mechanisms determine the lower prey size limit in suspending feeding copepods. Regarding the upper prey size, planktonic copepods and other crustaceans have strong mandibles that can break the prey (e.g., diatoms) before ingestion, allowing them to feed on particles larger than the mouth. Although copepods could potentially break some types of plastics, the mouth’s dimensions physically constrain the upper size limit of the MPs that can be ingested. Most of the copepods in our study have a prosome length of 0.5–1 mm and are expected to have a mouth size <100 μm.^42,60^ The size of MPs found inside field zooplankton samples is highly variable, ranging from 3 to 2485 μm, depending on the species/groups (Table S5). In the case of copepods, fibers can be ingested due to the thinner width, but, in some cases, the size of the reported ingested MPs fragments for copepods is larger than their mouths, suggesting either entanglement or sample contamination rather than ingestion.

The shapes of MPs can also influence their ingestion.^61^ MPs of different shapes, e.g., fibers and fragments) have been found in field-collected zooplankton samples. Washing textiles can release many thousands of fibers,^62−64^ and MP fragments and other shapes are also discharged through various sources, i.e., cosmetics^58^ or form secondary MPs. Fibers were the most often found shape of MPs in marine zooplankton according to several field studies in the Northeast Pacific, Northern South China Sea, and East China Sea.^28,32,46,65^ We also found ingestion of a substantial number of fibers (41–55%, depending on the samples) in the zooplankton samples, but fragments were dominant in the fecal pellet samples, suggesting a lower ingestion of polyester fibers and a higher risk of entanglement for fibers than for fragments.

Many of the polymers found in the water samples^13^ were also detected in the zooplankton and fecal pellets. Polyester was the prominent polymer in the zooplankton samples, followed by polypropylene, in agreement with other studies.^66^ Higher amounts of polyester, a major component of synthetic clothing materials, are found in laundry effluent.^63^ Other polymers such as cellophane, polyester (e.g., Bohai Sea^34^), and polyurethane (Fram strait^49^) have been found to be the most abundant polymers in zooplankton. However, the percentage of polyester was lower in the fecal pellet samples after blank corrections, suggesting a lower ingestion of polyester fibers in copepods, as explained above. Studies (e.g., Botterell et al., 2022;^49^ Sipps et al., 2022^33^, and our study) suggest the MP polymer composition in zooplankton is quite similar to the one in the water column. This shows that the MPs polymers encountered by zooplankton are probably a function of the MPs in the surrounding seawater^33^ and their accidental ingestion, without discrimination among polymer types, as observed in Xu et al., 2022.^53^

### 4.4 Ecological Implications

The biomass of natural prey, i.e., phytoplankton, was 3–4 orders of magnitude higher than MPs mass in the surveyed Danish marine waters,^13^ so negative physical effects of the ingested MPs on the zooplankton samples appear to be unlikely. Although MPs are not expected to be highly ingested by zooplankton, plastic leachates can still cause negative effects on marine planktonic organisms due to their toxicity.^67,68^

A large part of fecal pellets from small zooplankton are recycled in the water column by microbial decomposition and coprophagy.^69^ Accordingly, we found that, although fecal pellets contained around 5% of the total MPs in the studied surface water layer,^13^ only 1.4% were exported to the pycnocline. This suggests that the remaining pellets underwent degradation, leading to the release of MPs into the water column and reducing the flux of MPs to the benthos. Our results indicate that quantitative contribution of fecal pellets to the vertical exportation of MPs is lower compared to other processes like aggregation to detritus/marine snow.^69−72^ However, given the importance of zooplankton fecal pellets in the biological carbon pump, the ecological relevance of this process should not be neglected. It has been hypothesized and documented by some laboratory studies that the ingestion of MPs by zooplankton changes the sinking velocity of fecal pellets.^61,73,74^ In the natural environment, at the currently common concentrations of MPs found in marine waters and inside of fecal pellets, the impact of MPs on the sinking rates of fecal pellets is expected to be of minor importance and the biological carbon pump may not be disturbed. However, more field research is needed to quantify the importance of the “biological plastic pump” in the vertical transport of MPs in marine systems.^72,75,76^

Zooplankton clear large volumes of water for feeding,^77^ increasing the risk of MP ingestion. However, based on the available information, the occurrence and ingestion of MP ingestion seems to be higher in nektonic animals like marine mammals, sea birds, marine turtles, and fishes (e.g., Duncan et al. 2019;^78^ Kühn and Franeker, 2020^79^) than in zooplankton. As explained above, the low ingestion of MPs by zooplankton can be explained by their mechano- and chemosensorial mechanisms for detecting, capturing, and selecting prey. Some nektonic animals are less efficient in discriminating between normal food and MPs than planktonic copepods and have a high risk of accidental ingestion of MPs when feeding, with the occurrence of ingested MPs/plastic debris up to 100% in some cases (e.g., Duncan et al., 2019^78^). For example, in a global analysis, it was found that 49% of sampled fish for MP studies had ingested MPs, an average of 3.5 MPs per fish.^80^ Commonly ingested MPs are the same size as zooplankton (e.g., 300 to 500 μm, Cordova, et al, 2020^81^) or have similar colors,^82^ indicating that these MPs are directly ingested from the water and not via ingested MP-contaminated zooplankton.

Overall, our findings show a low ingestion of MPs down to 10 μm in zooplankton, suggesting that the risk of MPs transferring to higher trophic levels is lower compared to other pathways such us direct ingestion of MPs suspended in the water (e.g., Ory et al., 2017^82^) or via MP-contaminated marine snow.^70^ Therefore, zooplankton are an entry pathway of MPs into the food webs, but their quantitative contribution to transfer and vertical exportation of MPs in marine systems is expected to be lower than other physical and biological mechanisms. Given the key role of zooplankton and fecal pellets in marine ecological processes, more research is needed to evaluate how these biologically mediated pathways can influence the physical (particle itself) and chemical (associated additives and absorbed contaminants) impacts of plastic pollution in marine ecosystems.

## Supporting Information Available

Seven supplementary figures (Figures S1–S7) and five supplementary tables (Tables S1–S5) (PDF)
