# Supplementary material for: Is Zooplankton an Entry Point of Microplastics into the Marine Food Web?
Source: Environ Sci Technol. 2023 Jul 27;57(31):11643–55. doi: 10.1021/acs.est.3c02575 (PMC10413952; doi:10.1021/acs.est.3c02575)
Supplement: Supplementary file 1 — es3c02575_si_001.pdf [file es3c02575_si_001.pdf]

SUPPLEMENTARY INFORMATION

Is zooplankton an entry point of microplastics into the marine food web?

Kuddithamby Gunaalan[1,2*], Torkel Gissel Nielsen[1]; Rocío Rodríguez Torres[1,3], Claudia Lorenz[2], Alvise Vianello[2], Ceelin Aila Andersen[1], Jes Vollertsen[2], Rodrigo Almeda[1, 4*]

guku@aqua.dtu.dk; rodrigo.almeda@ulpgc.es

[1]National Institute of Aquatic Resource, Technical University of Denmark, Kemitorvet, 201, 2800 Kgs., Lyngby, Denmark

[2]Department of the Built Environment, Aalborg University, Thomas Manns Vej 23, 9220 Aalborg East, Denmark

[3]Laboratoire d'Océanographie de Villefranche sur mer (LOV), UPMC Université Paris 06, CNRS UMR 7093, Sorbonne Université, Villefranche sur Mer, France

[4]EOMAR-ECOAQUA, University of Las Palmas of Gran Canaria, 35017 Las Palmas de Gran Canaria, Spain.

* Corresponding Authors

CONTENTS:

- 15 pages/7 figures/ 5 tables
- Figure S1-S7
- Table S1-S5
- References

Supplementary Information

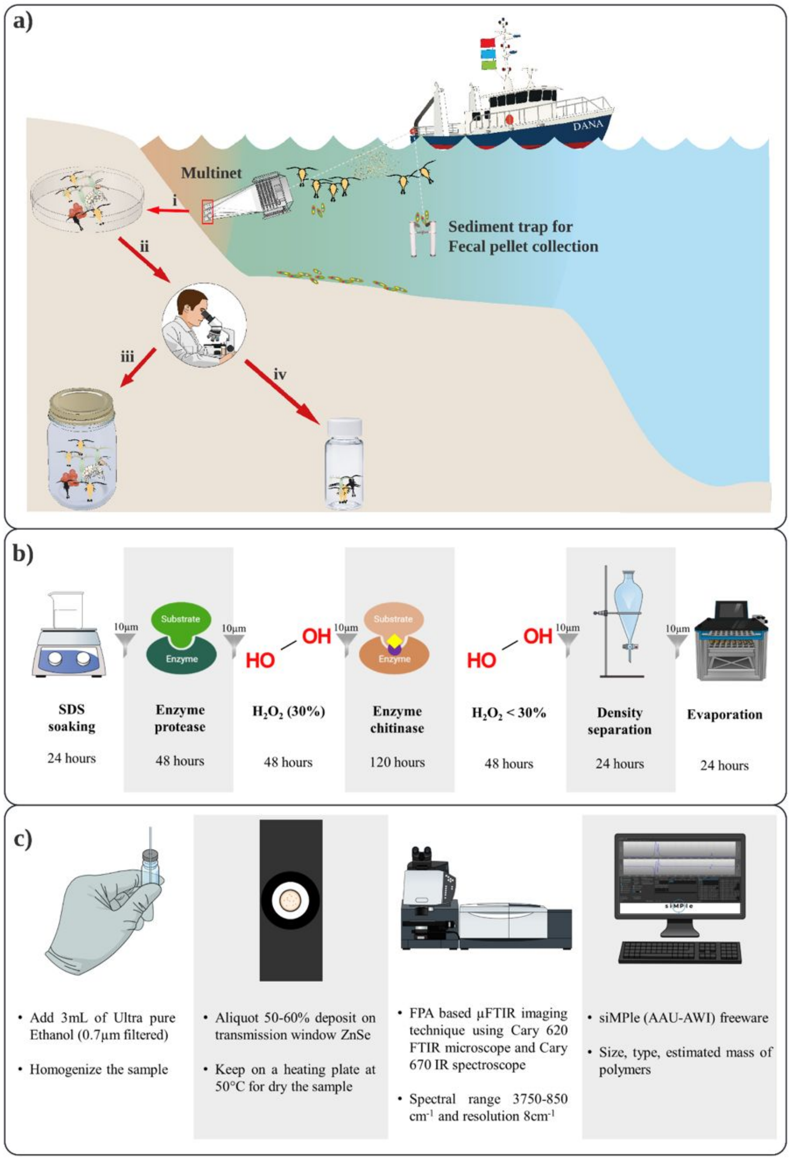

Figure S1: Sample collection (a), sample preparation (b), and MP detection methods (c) where, a) (i) zooplankton were collected using a multi net (MOCNESS), (ii) the zooplankton were examined with a stereomicroscope, (iii) the natural zooplankton communities at station 13 and 14 were stored in big glass jars along with SDS (iv) around 200 copepods were separated at each station and placed in 20mL glass vials along with SDS

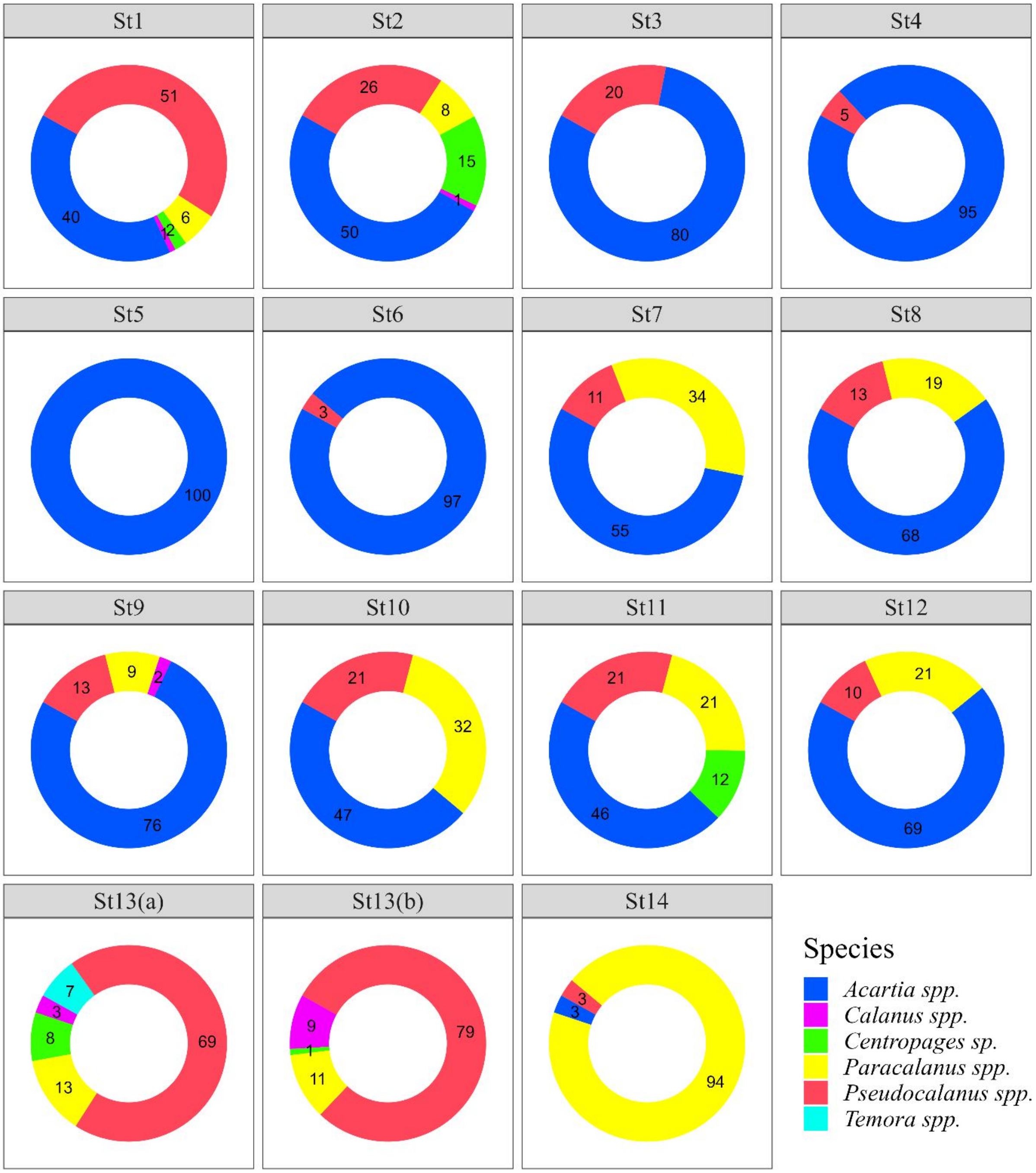

Figure S2: Percentage distribution of calanoid species in the study area and sorted for MP analyses.

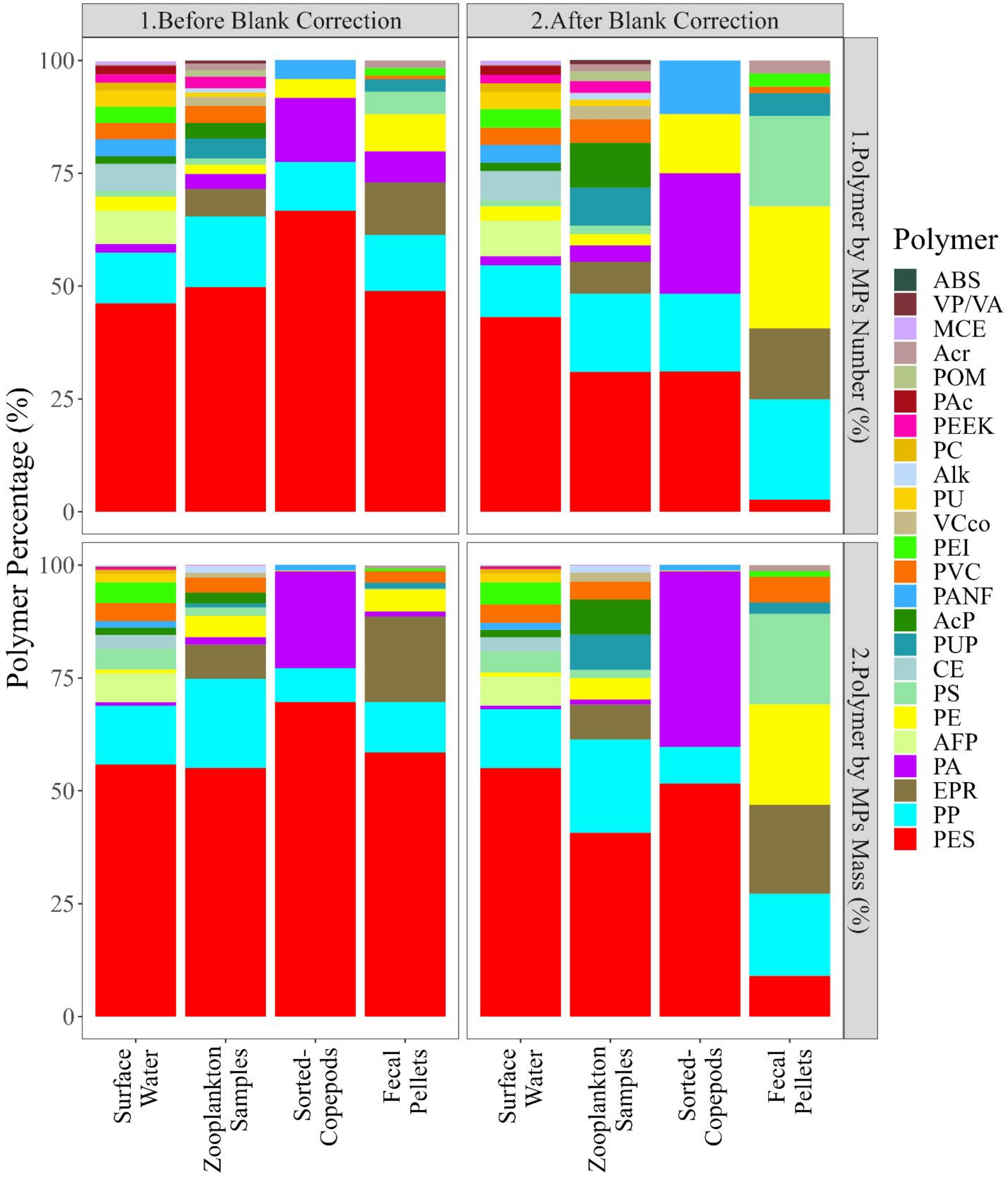

Figure S3: Average percentage of polymer composition among samples. The upper panel shows the percentage of polymer composition based on numbers and the lower panel illustrates the percentage of polymer composition based on mass estimates of the MPs before and after blank correction.

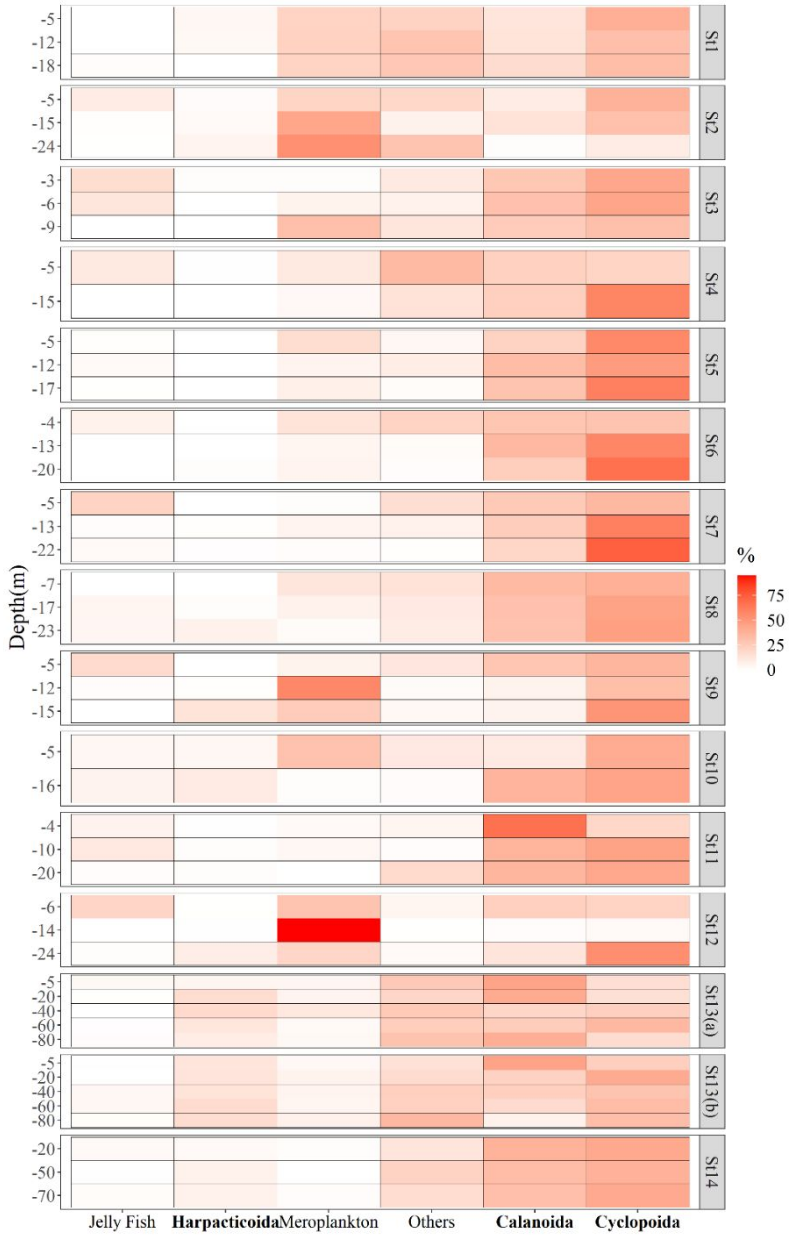

Figure S4: Zooplankton community composition in the study area, the copepod orders are bolded.

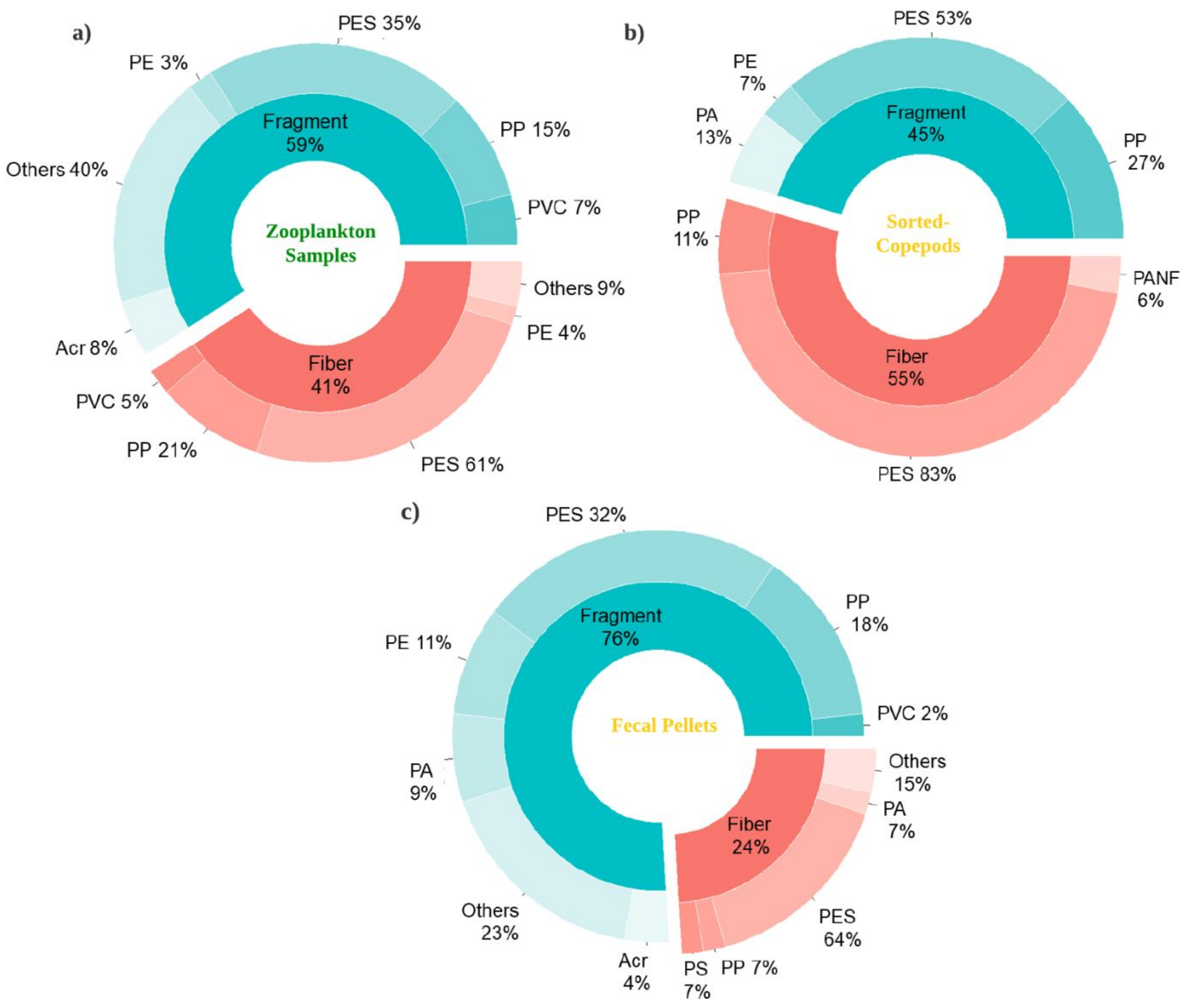

Figure S5: MPs particle shape (fiber/fragment) and polymer composition in natural zooplankton samples (a), sorted-copepods (b), and fecal pellets (c).

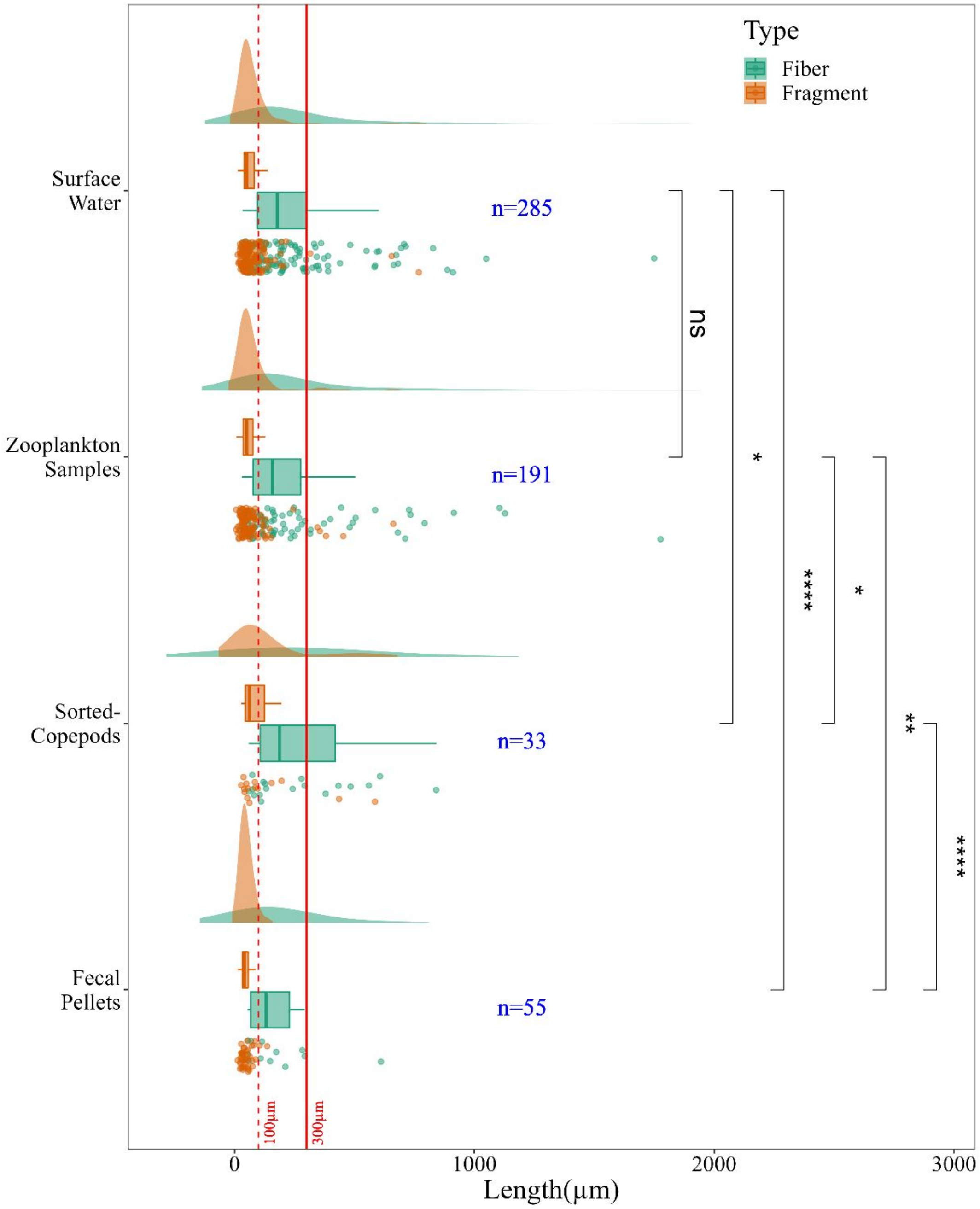

Figure S6: The length distribution of the MP fibers and fragments in the surface water[1], zooplankton samples, sorted-copepods, and fecal pellets in the study area. "Raincloud plots" (Allen et al., 2019)[2] visualize raw data (points), length-frequency distribution (density), key summary statistics of the median, interquartile range (boxplot) and Kruskal-Wallis test between

the lengths of samples where, ns: no significant, $p > 0.05$, *: $p \leq 0.05$, **: $p \leq 0.01$, ***: $p \leq 0.001$, ****: $p \leq 0.0001$

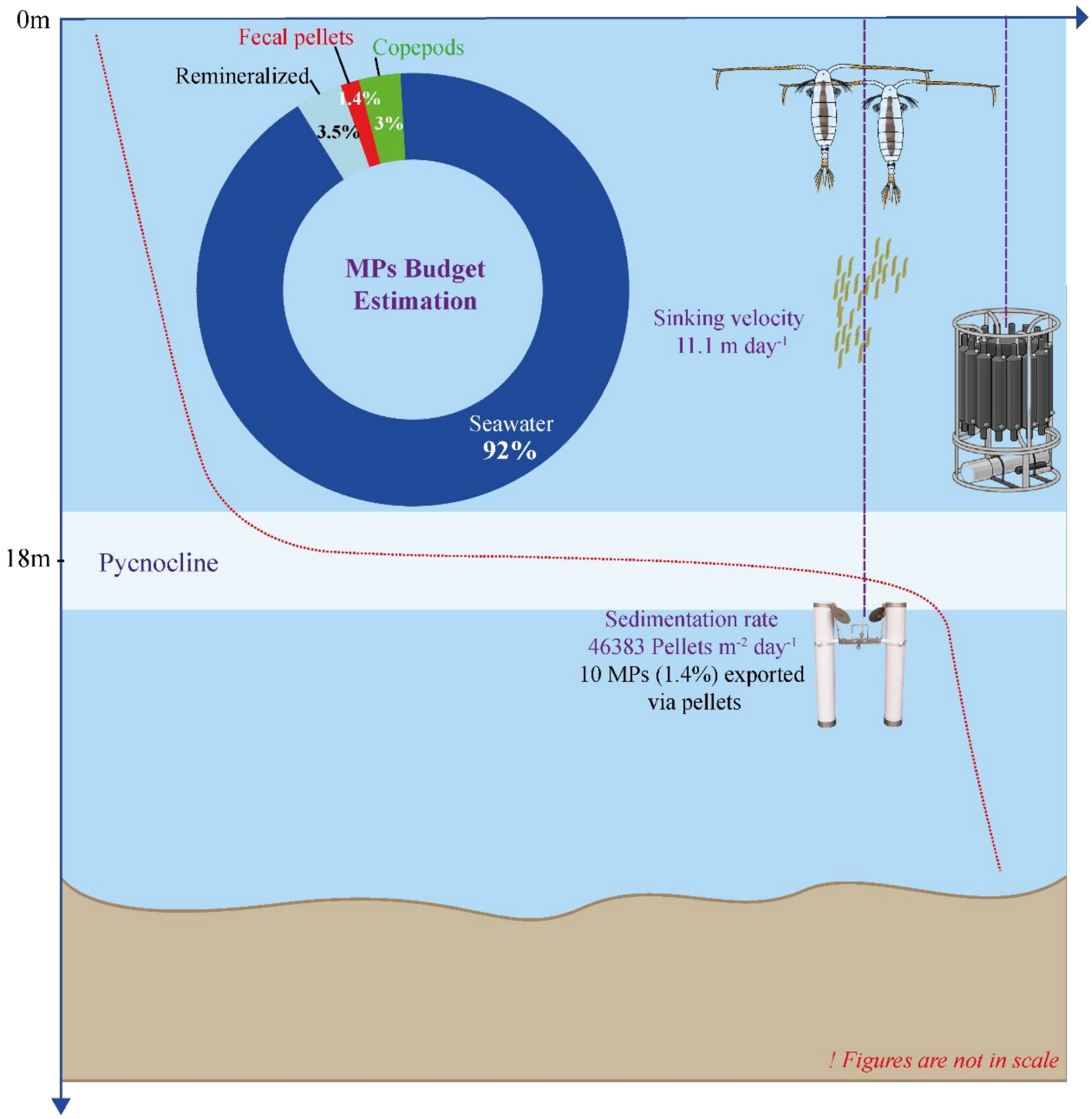

Figure S7: Schematic illustration of the estimated MPs budget for surface waters (18m) and exportation of MPs via fecal pellets

Table S1: Blank correction data for the natural zooplankton sorted copepods and fecal pellets

Number of working days	8
Number of working hours	192
Total MPs in air blank	819.23
MP/h	4.27
Natural zooplankton container area (cm^2)	63.58
Air blank Petri dish area (cm^2)	153.8
Sorted sample container (small Petri dish) area (cm^2)	19.64
**Air blank correction for Zooplankton Samples**	
hour of contamination (hour)	1
4.27 (MP/h)*(63.58cm^2/153.8cm^2)*1h	**1.76**
**Air blank correction for Pooled (Sorted Copepod & Fecal pellets)sample**	
hour of contamination (hours)	8
4.27 (MP/h)*(19.64cm^2/153.8cm^2)*8h per station	**4.36**
Zone I (st1,2)	8.72
Zone II (st3,7,8,9,12)	21.79
Zone III (st4,5,6)	13.07
Zone IV (st10,11)	8.72
Zone V (st13,14)	8.72
**MPs in ship workstation's water blank sample (FWB) and Lab blanks (LB)**	
FWB1	13.1
FWB2	3.8
FWB3	0.0
FWB4	9.4
FWB5	0.0
FWB6	5.6
FWB7	0.0
FWB8	6.7
LB1	38.0
LB2	20.0
**Median of FWB and LB**	**6.1**

Table S 2: Summary of MPs characteristics for the different samples of the study area

Medium	Type	Length(µm)		Width (µm)		Feret Minimum (µm)		Mass (µg)	
		Median	Mean±SD	Median	Mean±SD	Median	Mean±SD	Median	Mean±SD
**Surface Water[1] (n=285)**	Total MPs	79.6	151.3±196.5	27.6	36.4±37.2	44.6	65.8±72.2	0.025	0.738±6.545
	Fiber	178.3	248.2±248.4	26.8	37.4±41.9	57.3	85.2±92.6	0.059	1.229±9.424
	Fragment	51.7	74.4±84.6	27.6	35.5±33.0	38.5	50.3±45.1	0.015	0.348±2.525
**Zooplankton Community (n=191)**	Total MPs	72.7	149.5±220.5	25.95	38.5±43.3	39.3	68.6±101.0	0.021	0.670±3.269
	Fiber	154.4	255.9±296.1	26.6	41.4±44.2	44.6	94.7±134.6	0.036	0.961±3.630
	Fragment	51.7	74.6±89.2	25.8	36.4±42.8	33.0	50.2±62.4	0.014	0.464±2.988
**Sorted Copepods (n=33)**	Total MPs	110.9	212.8±210.4	32.9	52.3±55.5	49.5	96.7±113.6	0.050	1.007±2.899
	Fiber	187.6	278.3±227.2	30.05	45.4±43.2	52.8	107.2±127.3	0.055	0.748±1.508
	Fragment	62.3	134.1±162.2	37.9	60.6±68.2	44.6	84.0±97.6	0.025	1.318±4.032
**Fecal Pellets (n=55)**	Total MPs	54.6	77.9±91.0	23	29.4±22.6	33.0	43.0±38.9	0.011	0.155±0.928
	Fiber	113.5	169.2±150.1	21.9	35.5±38.4	37.2	68.1±69.2	0.029	0.551±1.882
	Fragment	40.8	48.8±24.2	23.0	27.5+14.6	33.0	35.0±16.9	0.009	0.029±0.065

Table S 1: Summary of MPs concentration in different compartments of the study area: * denotes the concentration of surface water MPs which is adapted from Gunaalan et al., 2023[1]

Compartment	MPs concentration (MPs ind^{-1} or MPs $pellet^{-1}$)	Number of individuals or fecal pellets per cubic meter	MPs concentration (MPs m^{-3})		%
Seawater			39.0*	35.9	92
Copepods	0.00095	1283	1.2	1.2	3
Fecal pellets	0.00022	8583	1.9	1.9	5

Table S 2: Summary of fecal pellet sedimentation rate, sinking velocity and pellets numbers in surface water at 5m depth (n=11)

	Sedimentation rate (Pellets m^{-2} day^{-1})	Pellets concentration in surface water at 5m depth (Pellets m^{-3})	Sinking velocity (m^{-1} day^{-1})	Sediment trap hanging depth (m)
Station 1	37280	4100	9.1	20
Station 2	57932	1000	57.9	20
Station 3	9607	1050	9.1	10
Station 4	24317	4600	5.3	15
Station 5	92036	27014	3.4	15
Station 6	65150	12450	5.2	20
Station 7	83353	10600	7.9	25
Station 9	24887	3000	8.3	15
Station 10	73307	8750	8.4	15
Station 11	16110	17950	0.9	15
Station 12	26238	3900	6.7	25
**Average**	**46383**	**8583**	**11.1**	**18**

Table S 3: Summary of previous studies on ingestion MPs by natural zooplankton community in marine environment

Organism	Sample Prep. method	MP detection method	Conc. MPs in zooplankton (MPs ind^{-1})	Conc. MPs in water (MPs m^{-3})	MP Size (µm)	Polymers/Type of particles	Location	Reference
Neocalanus cristatus	Strong acids	Visual	0.03		556 ± 149		Northeast Pacific Ocean	[3]
Euphausia pacifia			0.06		816 ± 108			
**Chaetognatha**	1M NaOH	ATR-FTIR	0.46	110	100-1600	LDPE dominant in zooplankton	Kenya	[4]
**Copepod**			0.33					
**Amphipod**			0.22					
**Fish larvae**			0.16					
**Copepod**	100% HNO3	Visual/ATR	0.13	19.7 ± 22.4	295.2 ± 348.6	Fiber	East China Sea	[5]
**Pteropod**			0.35		20.3 ± 11.0	Pellet		
					82.4 ± 80.5	Fragment		
**Fish larvae**	65% HNO_3	Visual/SEM	0.14	3300		PA fibers dominant in seawater	South China Sea	[6]
**Cyclopoid**			0.13		20			
**Shrimps**			0.01		1680			
**Polychaeta**			0.007					
**Calanoid**			0.005					
**Chaetognath**			0.003					
Temora longicornis	30% KOH & NaClO	Visual	Nil	8.2			Chichester Harbor, UK	[7]

Organism	Sample Prep. method	MP detection method	Conc. MPs in zooplankton (MPs ind^{-1})	Conc. MPs in water (MPs m^{-3})	MP Size (µm)	Polymers/Type of particles	Location	Reference
**Copepod**	30% H_2O_2 & 1M HCl	Visual	1.4% of zooplankton on have ingested microplastics		43-104		Charleston Harbor, SC USA	[8]
**Copepod Rainy season**	10% KOH	Visual and µFTIR	~0.025		1300 ± 1520	Cellophane(53%) Polyester (18)	Bohai Sea, China	[9]
**Dry season**			~0.005		1040 ± 1060	Cellophane(68%) Polyester (20%)		
**Copepod**	100% HNO_3	Visual and µFTIR	0.21 ± 0.10 (mean± se)		90 to 2485	Polyester, Cellophane	Jiaozhou Bay,China	[10]
Calanus euxinus	Wet peroxidation	Visual & FTIR	0.024 ± 0.020	0.12 to 7.62	100± 153	PET (21%), PA (11%), PP (7%), PE (6%) in water	Black Sea	[11]
Acartia clausi			0.008 ± 0.006		62 ± 56			
**Fecal pellets**			0.011		32 ± 12			
Themisto libellula	Enzymatic digestion	FTIR & siMPle freeware	1.8 ± 0.2	0 to 18,500	65.4 ± 16.8 (15–146)	Acrylic, PE, PS, PU, PVDC	Farm strait	[12]
T. abyssorum			1		64 ± 14.8 (33−113)	Acrylic, PE, Polyester		
Apherusa glaciali			1		31	Polyester		
Calanus hyperboreus			0.21 ± 0.03		26.2 ± 7.5 (8–286)	PS, PU		
C.glacialis/ finmarchicus			0.01 ± 0.003		58.2 ± 13.4 (8–158)	Acrylic, PS, PU		
Acartia tonsa,	70% HNO_3	µFTIR and/or Raman	0.30-0.73		Beads ϕ 5µm Films	PE, PP, PS, Epoxy resin, Polyester,	Hudson-Raritan estuary,	[13]
Paracalanus crassirostris			0.60-0.74					

Organism	Sample Prep. method	MP detection method	Conc. MPs in zooplankton (MPs ind^{-1})	Conc. MPs in water (MPs m^{-3})	MP Size (µm)	Polymers/Type of particles	Location	Reference
Centropages typicus		spectroscopic	0.82		7-60µm Fragments 3–165µm	PDMS	USA	
**Zooplankton samples**	Enzymatic-oxidative digestion (Chitinase was used)	FPA-µFTIR & siMPle freeware	0.002* (0.004±0.006)	11 to 87**	72.7*	Polyester (48%), PP (15%), PE (3%)	Kattegat/ Skagerrak, Denmark	Present study
**Sorted-copepods**			0.003* (0.02±0.03)		110.9*	Polyester (67%), PP (11%), PA (14%)		
**Fecal pellets**			0.0003* (0.009±0.014)		54.6*	Polyester (49%), PP (12%), PE (8%)		

*denotes the medians of the MPs size

** denotes the concentration of surface water MPs which is adapted from Gunaalan et al., 2023[1]

PDMS-Polydimethylsiloxane; PE-Polyethylene; LDPE- Low dense polyethylene; PP-Polypropylene; PA-Polyamide; PU-Polyurethane; PS-Polystyrene; PVDC- Polyvinylidene chloride

References:

(1) Gunaalan, K.; Almeda, R.; Lorenz, C.; Vianello, A.; Iordachescu, L.; Papacharalampos, K.; Rohde Kiær, C. M.; Vollertsen, J.; Nielsen, T. G. Abundance and Distribution of Microplastics in Surface Waters of the Kattegat/ Skagerrak (Denmark). *Environ. Pollut.* **2023**, *318*, 120853. https://doi.org/10.1016/j.envpol.2022.120853.

(2) Allen, M.; Poggiali, D.; Whitaker, K.; Marshall, T. R.; Kievit, R. A. Raincloud Plots: A Multi-Platform Tool for Robust Data Visualization. *Wellcome Open Res.* **2019**, *4*, 63. https://doi.org/10.12688/wellcomeopenres.15191.1.

(3) Desforges, J.-P. W.; Galbraith, M.; Ross, P. S. Ingestion of Microplastics by Zooplankton in the Northeast Pacific Ocean. *Arch. Environ. Contam. Toxicol.* **2015**, *69* (3), 320–330. https://doi.org/10.1007/s00244-015-0172-5.

(4) Kosore, C.; Ojwang, L.; Maghanga, J.; Kamau, J.; Kimeli, A.; Omukoto, J.; Ngisiag'e, N.; Mwaluma, J.; Ong'ada, H.; Magori, C.; Ndirui, E. Occurrence and Ingestion of Microplastics by Zooplankton in Kenya's Marine Environment: First Documented Evidence. *Afr. J. Mar. Sci.* **2018**, *40* (3), 225–234. https://doi.org/10.2989/1814232X.2018.1492969.

(5) Sun, X.; Liu, T.; Zhu, M.; Liang, J.; Zhao, Y.; Zhang, B. Retention and Characteristics of Microplastics in Natural Zooplankton Taxa from the East China Sea. *Sci. Total Environ.* **2018**, *640–641*, 232–242. https://doi.org/10.1016/j.scitotenv.2018.05.308.

(6) Md Amin, R.; Sohaimi, E. S.; Anuar, S. T.; Bachok, Z. Microplastic Ingestion by Zooplankton in Terengganu Coastal Waters, Southern South China Sea. *Mar. Pollut. Bull.* **2020**, *150*, 110616. https://doi.org/10.1016/j.marpolbul.2019.110616.

(7) Outram, L.; Hurley, J.; Rott, A.; Crooks, N.; Pernetta, A. P. No Evidence of Microplastic Consumption by the Copepod, *Temora Longicornis* (Müller, 1785) in Chichester Harbour, United Kingdom. *Nauplius* **2020**, *28*. https://doi.org/10.1590/2358-2936e2020033.

(8) Payton, T. G.; Beckingham, B. A.; Dustan, P. Microplastic Exposure to Zooplankton at Tidal Fronts in Charleston Harbor, SC USA. *Estuar. Coast. Shelf Sci.* **2020**, *232*, 106510. https://doi.org/10.1016/j.ecss.2019.106510.

(9) Zheng, S.; Zhao, Y.; Liangwei, W.; Liang, J.; Liu, T.; Zhu, M.; Li, Q.; Sun, X. Characteristics of Microplastics Ingested by Zooplankton from the Bohai Sea, China. *Sci. Total Environ.* **2020**, *713*, 136357. https://doi.org/10.1016/j.scitotenv.2019.136357.

(10) Zheng, S.; Zhao, Y.; Liu, T.; Liang, J.; Zhu, M.; Sun, X. Seasonal Characteristics of Microplastics Ingested by Copepods in Jiaozhou Bay, the Yellow Sea. *Sci. Total Environ.* **2021**, *776*, 145936. https://doi.org/10.1016/j.scitotenv.2021.145936.

(11) Aytan, U.; Esensoy, F. B.; Senturk, Y. Microplastic Ingestion and Egestion by Copepods in the Black Sea. *Sci. Total Environ.* **2022**, *806*, 150921. https://doi.org/10.1016/j.scitotenv.2021.150921.

(12) Botterell, Z. L. R.; Bergmann, M.; Hildebrandt, N.; Krumpen, T.; Steinke, M.; Thompson, R. C.; Lindeque, P. K. Microplastic Ingestion in Zooplankton from the Fram Strait in the Arctic. *Sci. Total Environ.* **2022**, *831*, 154886. https://doi.org/10.1016/j.scitotenv.2022.154886.

(13) Sipps, K.; Arbuckle-Keil, G.; Chant, R.; Fahrenfeld, N.; Garzio, L.; Walsh, K.; Saba, G. Pervasive Occurrence of Microplastics in Hudson-Raritan Estuary Zooplankton. *Sci. Total Environ.* **2022**, *817*, 152812. https://doi.org/10.1016/j.scitotenv.2021.152812.
